# Treatment of Obstructed Labour

**Published:** 1902-07-26

**Authors:** 


### TREATMENT OF OBSTRUCTED LABOUR.

At the last meeting of the Obstetrical Society a paper by Mr. J. W. Taylor brought up an interesting discussion on the relative advantages of the vaginal and abdominal routes for the removal of certain ovarian tumours obstructing labour. In the case reported delivery at full term had been obstructed by the presence of an ovarian tumour in the pelvis, and after considering the relative advantages of abdominal and vaginal ovariotomy it had been decided to make a posterior vaginal section and to perform ovariotomy by this route during the progress of labour. This was done, delivery was then accomplished without delay by the aid of forceps, and the patient had made an easy and satisfactory recovery. Commenting on the case, Dr. Peter Horrocks (the president) said that he could not help feeling that Mr. Taylor had been lucky in finding the ovarian cyst so simple and free from adhesions. Had it been multilocular with a large number of small cysts, or had there been a lot of adhesions to the bowel and other parts, or had the pedicle been difficult to reach, he might have been obliged to open the abdomen after all. One great danger of the vaginal route was the risk of the pedicle being torn in being pulled down within reach. He had tried both ways, and he must say that he preferred to tackle these tumours through the abdominal wall. Dr. Spencer thought that abdominal was preferable to vaginal ovariotomy in these cases. It could not be too strongly insisted upon that it was not necessary to do Cæsarian section in order to get at the imprisoned tumour by the abdominal route. In the case related, the tumour, which he understood contained two or three pints, must certaintly have reached above the brim of the pelvis, and might have been opened there, possibly without much difficulty; but in any case by enlarging the incision and turning out the uterus the tumour could have been easily dealt with, and then forceps could have been applied. The vaginal operation seemed to him to be inferior, in that it made a wound in the vagina, but did not permit inspection of the other ovary, or allow such careful treatment of adhesions or of the pedicle as could be attained by abdominal section. Mr. Taylor had evidently found difficulty with the pedicle, and had had to cut the ligature short by the sense of touch. Cases had been recorded of slipping of the ligature, such cases showing the necessity of careful separate ligation of the ovarian vessels in the pedicle in cases of ovariotomy during pregnancy, and this he considered could only be satisfactorily performed by the abdominal operation. Mr. Taylor in reply said that the operation he performed was deliberately chosen as the outcome of an experience of vaginal cœliotomy now extending over some nine or ten years. It was only in the minority of cases (some 5 per cent. possibly) that an operation begun by the vagina had to be finished by the abdominal route, and even when this was necessary he had found that the additional vaginal opening was no disadvantage, being often exceedingly useful for drainage. Undoubtedly the chief difficulty of the vaginal operation was the comfortable securing of the pedicle, but the surgeon could leave a pair of forceps on the stump with perfect safety if necessary. The open method of treating the vaginal wound, with an iodoform gauze drain, also precluded the possibility of serious hæmorrhage occurring without the immediate knowledge of those in attendance. He could not help contrasting the operation described by Dr. Spencer with the simple, direct, and comparatively easy method employed in his own case. As another advantage in the proceeding which he had adopted, he pointed to the fact that the patient recovered without any abdominal wound or cicatrix.

